# Investigating the clinical factors and comedications associated with circulating levels of atorvastatin and its major metabolites in secondary prevention

**DOI:** 10.1111/bcp.14133

**Published:** 2020-01-04

**Authors:** Richard M. Turner, Vanessa Fontana, Richard FitzGerald, Andrew P. Morris, Munir Pirmohamed

**Affiliations:** ^1^ Department of Molecular & Clinical Pharmacology University of Liverpool United Kingdom; ^2^ Department of Biostatistics University of Liverpool United Kingdom

**Keywords:** drug–drug interactions, loop diuretics, myotoxicity, proton pump inhibitors, smoking, statin, systemic exposure

## Abstract

**Aims:**

The lipid‐lowering drug, atorvastatin (ATV), is 1 of the most commonly prescribed medications worldwide. The aim of this study was to comprehensively investigate and characterise the clinical factors and comedications associated with circulating levels of ATV and its metabolites in secondary prevention clinical practice.

**Methods:**

The plasma concentrations of ATV, 2‐hydroxy (2‐OH) ATV, ATV lactone (ATV L) and 2‐OH ATV L were determined in patients 1 month after hospitalisation for a non‐ST elevation acute coronary syndrome. Factors were identified using all subsets multivariable regression and model averaging with the Bayesian information criterion. Exploratory genotype‐stratified analyses were conducted using *ABCG2* rs2231142 (Q141K) and *CYP2C19* metaboliser status to further investigate novel associations.

**Results:**

A total of 571 patients were included; 534 and 37 were taking ATV 80 mg and 40 mg daily, respectively. Clinical factors associated with ATV and/or its metabolite levels included age, sex, body mass index and CYP3A inhibiting comedications. Smoking was newly associated with increased ATV lactonisation and reduced hydroxylation. Proton pump inhibitors (PPIs) and loop diuretics were newly associated with modestly increased levels of ATV (14% and 38%, respectively) and its metabolites. An interaction between PPIs and *CYP2C19* metaboliser status on exposure to specific ATV analytes (e.g. interaction *P* = .0071 for 2‐OH ATV L) was observed. Overall model R^2^ values were 0.14–0.24.ConclusionMultiple factors were associated with circulating ATV and metabolite levels, including novel associations with smoking and drug–drug(–gene) interactions involving PPIs and loop diuretics. Further investigations are needed to identify additional factors that influence ATV exposure.

**What is already known about this subject**

- Atorvastatin is a well‐tolerated hypolipidaemic drug, but leads to myotoxicity in a subset of patients.
- Factors known to increase systemic statin exposure such as increased statin dose, and concurrent use of CYP3A inhibiting drugs, predispose to statin myotoxicity.

**What this study adds**

- Smoking was newly associated with increased atorvastatin lactonisation and decreased hydroxylation.
- Proton pump inhibitors and loop diuretics were newly associated with modestly increased exposure to atorvastatin and its metabolites.
- A putative interaction between proton pump inhibitors and *CYP2C19* metaboliser status on exposure to specific atorvastatin analytes was observed.

## 1 INTRODUCTION

Statins are amongst the most highly prescribed medications worldwide. https://www.guidetopharmacology.org/GRAC/LigandDisplayForward?ligandId=2949 (ATV) is currently the guideline‐recommended first‐line hypolipidaemic drug for primary and secondary prevention of cardiovascular (CV) disease (CVD). Statins reduce major CV events by ~20–30% per 1.0 mmol/L reduction in low‐density lipoprotein –cholesterol (LDL‐C), across a range of baseline CV event risks.^1^ Whilst efficacious and generally well tolerated, statins can cause adverse effects in a small proportion of patients including incident diabetes mellitus^2^ and statin‐associated myotoxicity (SAM).^3^

**Figure 1:**
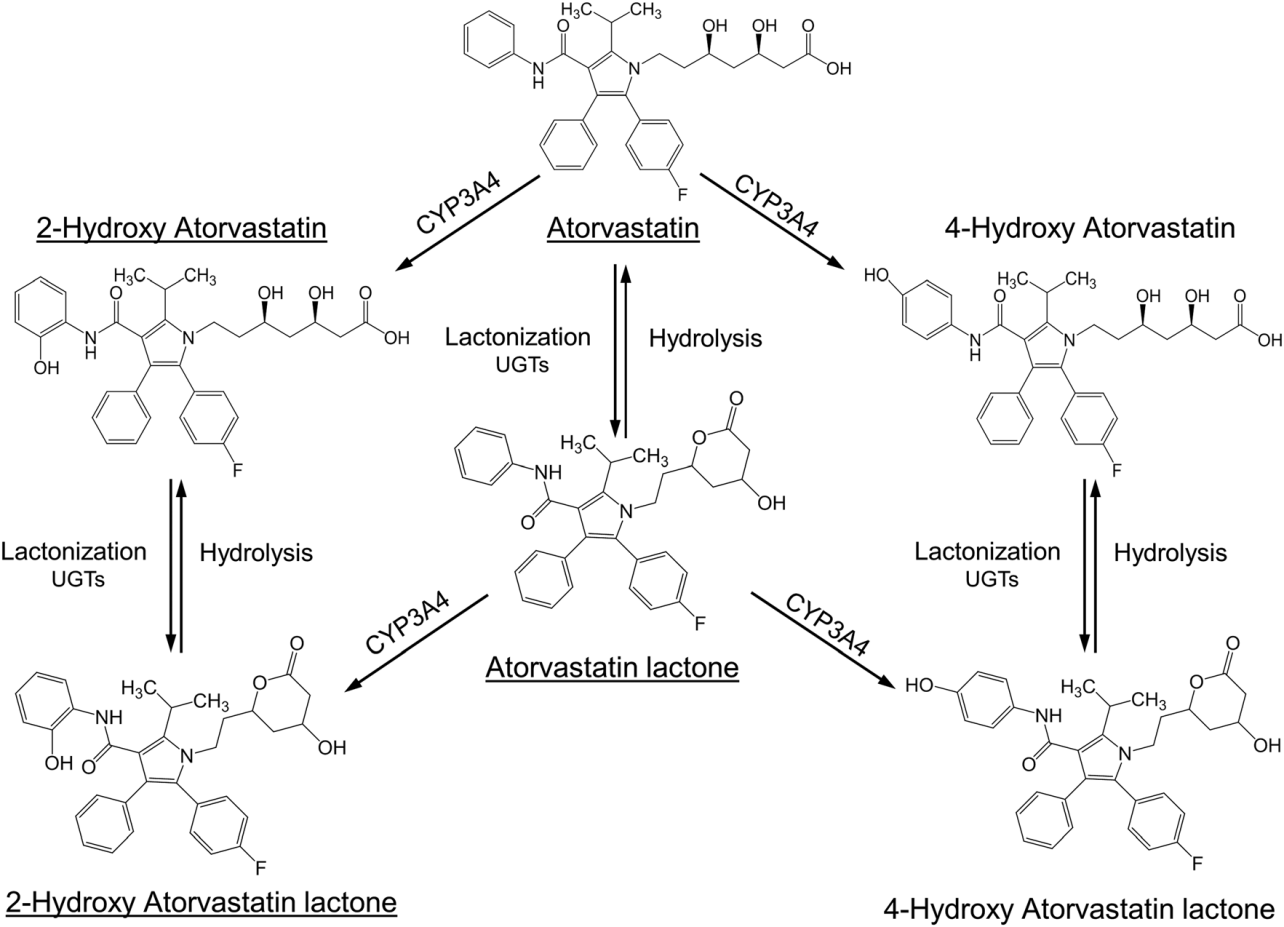
The biotransformation pathway of atorvastatin. CYP3A4 = cytochrome P450 3A4; UGTs = uridine 5′‐diphospho‐glucuronosyltransferases. The major analytes are underlined and were investigated here

ATV is administered as a calcium salt of its active carboxylic acid form^4^ and its metabolism is shown in Figure 1. Briefly, ATV can undergo hydroxylation and/or lactonisation, mediated principally by cytochrome P450 3A4 (https://www.guidetopharmacology.org/GRAC/ObjectDisplayForward?objectId=1337)^5^ and uridine 5′‐diphospho‐glucuronosyltransferases (UGTs),^6^ respectively. ATV and its acid metabolites, https://www.guidetopharmacology.org/GRAC/LigandDisplayForward?ligandId=6705 and 4‐OH ATV, all inhibit 3‐hydroxy‐3‐methyl‐glutaryl‐coenzyme A reductase (https://www.guidetopharmacology.org/GRAC/ObjectDisplayForward?objectId=639) to lower LDL‐C. All 3 lactone (L) metabolites (https://www.guidetopharmacology.org/GRAC/LigandDisplayForward?ligandId=2957, 2‐OH ATV L, 4‐OH ATV L) are inactive against HMGCR but can be hydrolysed both nonenzymatically and via plasma esterases and paraoxonases to their corresponding acids.^7^, ^8^ ATV and its metabolites are eliminated predominantly via the biliary system.^4^

All statins, including ATV, can result in SAM, which ranges from common myalgias (in ~5% patients^9^) where causal attribution may be difficult, through to myopathies of increasing severity with elevated plasma creatine kinase levels (~0.1% patients) to rare rhabdomyolysis (0.1–8.4/100 000 patient‐years).^3^ Although the mechanisms remain incompletely understood, factors that increase systemic statin exposure appear to increase the risk of SAM. These factors include higher statin dose, advanced age, low body mass index (BMI), and comedications that inhibit CYP3A4 in patients on a statin that is a CYP3A4 substrate (ATV, simvastatin lovastatin).^3^, ^10^, ^11^ Statin lactones are considered more myotoxic than statin acids.^12^

The aim of this work was to determine the plasma levels of ATV, and importantly its main acid and lactone metabolites, in a large cohort of patients on high dose ATV following a non‐ST elevation acute coronary syndrome (NSTE‐ACS), and to identify the clinical factors associated with these levels. To the best of knowledge, a real‐world assessment of ATV levels on this scale has not been previously reported.

## 2 METHODS

### 2.1 PhACS study

This investigation harnessed the Pharmacogenetics of Acute Coronary Syndrome (PhACS) study, described previously.^13^ Briefly, PhACS was a UK multicentre prospective observational study that recruited 1470 eligible patients hospitalised with an NSTE‐ACS between 2008–2012 and followed them up at 1 (V2) and 12 (V3) months. Demographic, comorbidity and prescription information was collected at baseline; drug use, drug adherence, and new clinical events were captured during follow up. PhACS participants were genotyped using the Illumina HumanOmniExpressExome‐8 v1.0 BeadChip at Edinburgh Genomics (Roslin Institute, Scotland).

The protocol was approved by the Liverpool (adult) research Ethics Committee, UK; site‐specific approval was granted at all sites involved and local informed consent was obtained from all study subjects in accordance with the Declaration of Helsinki.

### 2.2 Determination of ATV and metabolite plasma concentrations

The concentrations of ATV and its 3 main metabolites (2‐OH ATV, ATV L and 2‐OH ATV L)^14^ were determined in EDTA plasma samples collected at V2 by high‐performance liquid chromatography–tandem mass spectrometry, using an assay validated to European Medicines Agency criteria, as previously reported.^15^ The plasma aliquots used here had not been used before, minimising freeze–thaws. Briefly, 50‐μL plasma samples were first mixed with 20 μL of a composite internal standard solution containing a deuterated internal standard for each analyte, and then diluted further by addition of 180 μL 100% acetonitrile containing 0.3% acetic acid. Following centrifugation, the supernatant was diluted 1:2 in water and a 5‐μL aliquot injected for analysis. Analytes were separated using a 2.7‐μm Halo C18 column (50 × 2.1 mm ID, 90 Å, Hichrom Limited, Reading, UK, part number: 92812–402) with gradient elution, housed within a Shimadzu Nexera X2 modular system (Kyoto, Japan). Analytes were detected in low mass positive ionisation mode using multiple reaction monitoring (Sciex triple quadrupole 6500 QTRAP mass spectrometer with a Turbo electrospray source, AB Sciex, Warrington, UK). The ATV analyte transitions were (all [M + H^+^] *m/z*): ATV 559.3 → 440.3, 2‐OH ATV 575.3 → 250.2, ATV L 541.3 → 276.2, 2‐OH ATV L 557.3 → 276.2, ATV‐d5 564.1 → 445.1, 2‐OH ATV‐d5 580.2 → 255.1, ATV‐d5 L 546.3 → 281.2, 2‐OH ATV‐d5 L 562.2 → 281.1. Calibration and quality control (QC) samples were prepared in pooled healthy human volunteer K3 EDTA plasma (Sera Laboratories International, West Sussex, UK). The dynamic range of the ATV analytes utilised was 0.5–125 ng/mL.

### 2.3 Cohort selection

The inclusion criteria for this study were: all PhACS participants on 80 mg ATV daily at both baseline and V2, or on ATV 40 mg at both baseline and V2, and both drug adherence data and a stored plasma EDTA sample available at V2.

Four exclusion criteria were applied: (i) participants that failed standard genetic QC procedures^16^, ^17^; (ii) participants that had 2 or more ATV analyte levels <0.5 ng/mL (i.e. less than the uniform lower limit of quantification); (iii) participants with ATV concentrations ≥31 ng/mL (80 mg ATV) or ≥ 15.5 ng/mL (40 mg ATV), and; (iv) participants missing any data in the clinical variables considered (see below).

Exclusion criterion (i) was applied because, although a genome‐wide association study was not conducted here, adherence to its QC steps should improve the quality of the clinical data used (e.g. excluding participants with discordant clinical *vs* X‐chromosome‐derived sex) and would facilitate reliable use of specific single nucleotide polymorphisms selected for the genotype‐stratified analyses (see below). The PhACS genetic QC and imputation steps have been reported before.^17^ Participants with ≥2 analytes <0.5 ng/mL (exclusion criterion (ii)) were excluded because they were significantly more likely to be statin nonadherent (self‐reported to have missed at least 1 statin pill in the preceding week, *P* = .001) compared to participants with no low ATV analyte concentrations. However, those with only 1 analyte <0.5 ng/mL were not statin nonadherent (*P* = .88), might be exhibiting unorthodox ATV disposition/metabolism, and so were retained. As adherence data had been used to help select the cohort, it was not used in subsequent modelling. Exclusion criterion (iii) was applied because, whilst sample collection time was known, time of last ATV administration was not; time since last ATV was estimated by assuming all participants took their last ATV dose at 2200 the previous evening. A ceiling of 31 ng/mL was set, based on the mean ATV concentration plus 3 standard deviations (SDs) for ATV 80 mg from a previous ATV pharmacokinetics patient study^18^ to exclude participants with morning administration and near C_max_ levels; this ceiling concentration was halved for ATV 40 mg patients. Exclusion criterion (iv) was applied to ensure a complete dataset so all models produced for a given endpoint could be fairly compared.

### 2.4 Endpoints

The following 8 endpoints were considered: individual concentrations of ATV and its 3 metabolites, specific analyte ratios (hydroxylation—2‐OH ATV/ATV, 2‐OH ATV L/ATV L; lactonization—ATV L/ATV), and the analyte sum total (ATV + 2‐OH ATV + ATV L + 2‐OH ATV L).

### 2.5 Clinical variables

To characterise the cohort, baseline demographics and comorbidities, V2 drug status and blood sampling conditions were described as follows: ATV dose, sex, age, BMI, smoking (current *vs* previous/never), hypertension, hyperlipidaemia, diabetes mellitus, chronic kidney disease (CKD, defined as a serum creatinine >150 μmol/L), previous CVD (a myocardial infarction (myocardial infarction), stroke, transient ischaemic attack (TIA) or peripheral artery disease (PAD) before the index NSTE‐ACS), hepatic disease (a diagnosis of hepatic disease and/or history of alcohol excess), aspirin, a P2Y_12_ inhibitor, a β‐blocker, an angiotensin‐converting enzyme inhibitor/angiotensin II receptor blocker (ACEI/ARB), a loop diuretic, a thiazide diuretic, amiodarone, a proton pump inhibitor (PPI), on a CYP3A inducer, on a moderate or strong CYP3A inhibitor (supplementary Table S1 lists the CYP3A‐modifying drugs considered), the estimated time since last ATV dose (see above), and sample storage duration. All variables were considered in model selection (see below) except hypertension, hyperlipidaemia, and prior CVD.

### 2.6 Statistical analysis

Continuous variables are described by their mean (SD) or median (interquartile range) depending on the normality of their distribution, and categorical variables as their frequency (percentage).

As expected, raw ATV analyte concentrations showed a right‐skew distribution, and so the correlation between ATV analyte (untransformed) concentrations was assessed by Spearman's rank correlation. Prior to modelling, all endpoints were log_10_ transformed to normalise the distribution of (observed) residuals and optimise homoscedasticity. Clinical covariate multicollinearity was assessed using the variance inflation factor, which was <1.5 for all covariates, indicating negligible multicollinearity.

Multivariable linear regression was used for covariate selection, as has been done previously with sparse ATV sampling.^18^ For a given endpoint, first the maximum multivariable linear regression model (containing all 20 covariates) was constructed. Second, all subsets linear regression was performed from this maximum multivariable model, limited by the condition that all derived models include the time since last ATV dose covariate. Third, the derived models were ranked according to the Bayesian information criterion (BIC), which is more conservative than the corrected Akaike information criterion because BIC carries a larger penalty for increasing model complexity.^19^ Fourth, all models with a BIC within 2 of the model with the lowest BIC (BIC Δ < 2) underwent model averaging to produce model averaged (unstandardised) coefficients and standard errors for all the covariates included in this set of models, to enable multimodel inferences. The model selection constraint of Δ < 2 was chosen because the empirical evidence supporting each of these models is considered essentially equivalent.^19^ Conditional averaging was used, and so for a given covariate, its coefficient and standard error were averaged from only those models with BIC Δ < 2 that contained the covariate.^20^ This approach was taken because of the interest in assessing the relationship between individual covariates and the endpoints, with overall model predictive capability of secondary importance.^21^ The relative *importance* of each of the selected covariates to the endpoint was assessed by summing the BIC‐derived model weight for all models that underwent model averaging and contained the covariate. The *P*‐value for each covariate in the final model is provided for reference only. The utility of the final produced model for describing the observed variation in the endpoint was assessed using the unadjusted R^2^ value. This process was undertaken for all endpoints. The above described covariate selection and model averaging was carried out using the R statistical package, *MuMIn.* 20

#### 2.6.1 Secondary analysis

To compare model selection approaches, a second analysis was undertaken using multivariable linear regression with stepwise covariate selection based on the probability of F. A covariate was included in a step if its probability of F was both the lowest and <0.05, and previously selected covariates were removed if their probability became >0.10.

#### 2.6.2 Sensitivity analyses

To investigate the smoking covariate and identified drug–drug interactions further, patients with endpoint values outside the mean ± 2SDs were excluded and the multivariable model re‐tested. Secondly, novel plausible associated drug classes were substituted for 1 or more individual drugs from the class, and the multivariable models re‐run.

#### 2.6.3 Genotype‐stratified analyses

Exploratory genotype‐stratified analyses were carried out to further assess key identified drug–drug interactions. Specifically, the adjusted association of PPIs with endpoints was determined following cohort stratification by *CYP2C19* predicted metaboliser status, because PPIs are metabolised by CYP2C19.^22^, ^23^ *CYP2C19* was stratified by *2 (rs4244285; directly genotyped) and *17 (rs12248560; imputed) diplotypes into: extensive (EM, *1/*1), intermediate (IM, *1/*2 or *2/*17), poor (PM, *2/*2), rapid (RM, *1/*17) and ultra‐rapid metabolisers (UM, *17/*17). Due to the small number of PM (*n* = 10) and UM (*n* = 24) patients, PMs were combined with IMs, and UMs with RMs, for analysis. Only 1 individual was suggested to be a CYP2C19*3 (rs4986893) carrier, and so this variant was not considered further. Secondly, the impact of https://www.guidetopharmacology.org/GRAC/ObjectDisplayForward?objectId=792 rs2231142 (Q141K; imputed) on the adjusted associations of loop diuretics with the endpoints was considered. This is because loop diuretics (https://www.guidetopharmacology.org/GRAC/LigandDisplayForward?ligandId=4839, bumetanide) inhibit breast cancer resistance protein *in vitro* (BCRP, encoded by *ABCG2*),^24^, ^25^ and the rs2231142 minor allele is a reduction‐of‐function variant^14^; thus a BCRP‐mediated loop diuretic‐ATV interaction may be attenuated in 141 K carrier patients.

The sensitivity and genotype‐stratified analyses were only conducted on those endpoints where the perpetrator comedication of interest had been included in the endpoint's main analysis model, and *P* < .05 was taken to indicate significance. All statistical testing was carried out in the R computing environment,^26^ except for the secondary stepwise analysis that was undertaken in IBM SPSS version 22.0 (IBM Corp, Armonk, NY, USA); boxplots were produced in GraphPad Prism (version 5.00 for Windows: GraphPad Software Inc., San Diego, USA).

### 2.7 Nomenclature of targets and ligands

Key protein targets and ligands in this article are hyperlinked to corresponding entries in http://www.guidetopharmacology.org, the common portal for data from the IUPHAR/BPS Guide to PHARMACOLOGY.

## 3 RESULTS

The study cohort included 571 patients; 534 and 37 were prescribed ATV 80 and 40 mg daily, respectively. Table 1 reports the cohort's clinical characteristics; supplementary Figure S1 outlines the study cohort selection process. In keeping with a cohort of NSTE‐ACS patients, the majority were male with a high prevalence of cardiometabolic comorbidities and concomitant use of CVD secondary prevention medications. The plasma concentrations of the analytes displayed dose‐dependency, and were moderate to highly positively correlated with each other (r_s_ 0.47–0.83, Table 2). Interestingly, 2‐OH ATV and ATV L were the least correlated pair (r_s_ = 0.47), plausibly reflecting the different competing metabolic routes (hydroxylation *vs* lactonisation) available to ATV (Table 2).

**Table 1:** Cohort clinical characteristics

Characteristic	ATV
Number of patients	571
ATV dose:	
80 mg	534 (93.5%)
40 mg	37 (6.5%)
**Demographics:**	
Male	443 (77.6%)
Age, mean ± SD (years)	63.5 ± 11.5
Body mass index, median (IQR) (kg/m^2^)	28.2 (25.2–31.4)
Smoking:	
Previous/never	414 (72.5%)
Current	157 (27.5%)
**Comorbidities:**	
Hypertension	314 (55.0%)
Hyperlipidaemia prior to index NSTE‐ACS	295 (51.7%)
Diabetes mellitus	102 (17.9%)
CKD (Cr > 150 μmol/L)	32 (5.6%)
Prior CVD (previous MI, stroke, TIA or PAD)	168 (29.4%)
Hepatic disease	5 (0.9%)
**Drugs at visit 2:**	
Aspirin	539 (94.4%)
P2Y_12_ inhibitor	486 (85.1%)
Beta‐blocker	488 (85.5%)
ACEI/ARB	481 (84.2%)
Loop diuretic	96 (16.8%)
Thiazide diuretic	19 (3.3%)
Amiodarone	7 (1.2%)
Proton pump inhibitor	229 (40.1%)
CYP3A inducer	3 (0.5%)
Moderate/strong CYP3A inhibitor	21 (3.7%)
**Blood sample characteristics:**	
Sample storage duration, median (IQR) (y)	5.9 (5.3–7.4)
Time since last ATV, median (IQR) (h)	13.4 (12.5–15.8)

ACEI/ARB, angiotensin‐converting enzyme inhibitor/angiotensin II receptor blocker; ATV, atorvastatin; CKD, chronic kidney disease; CVD, cardiovascular disease; IQR, interquartile range; MI, myocardial infarction; NSTE‐ACS, non‐ST elevation acute coronary syndrome; PAD, peripheral artery disease; SD, standard deviation; TIA, transient ischaemic attack.

**Table 2:** Atorvastatin (ATV) analyte concentrations and correlations

Analyte	Conc (ng/mL), median (IQR)		Correlation (r_s_)			
	40 mg ATV	80 mg ATV	ATV	2‐OH ATV	ATV L	2‐OH ATV L
**ATV**	3.96 (1.91–7.92)	5.22 (2.96–9.66)	x	0.79	0.79	0.68
**2‐OH ATV**	5.35 (2.79–7.94)	6.93 (4.13–11.35)	0.79	x	0.47	0.66
**ATV L**	2.51 (1.05–5.39)	3.97 (2.22–7.48)	0.79	0.47	x	0.83
**2‐OH ATV L**	4.24 (2.70–7.13)	7.00 (4.37–11.88)	0.68	0.66	0.83	x

Tables 3 and 4 present the main analysis multivariable model selection results. For each endpoint, the individual models that were averaged and their model rank (BIC and corrected Akaike information criterion) are available in the supporting information (Tables S2 and S3). In general, the relative importance of ATV dose, demographics, comorbidities and blood sample characteristics in the models was high, whereas the importance of comedications was more variable. ATV dose was positively associated with the concentration of each ATV analyte and their total, but not with analyte ratios. Time since last ATV was strongly inversely correlated with analyte and total concentrations, but positively associated with 2‐OH ATV/ATV and ATV L/ATV ratios, indicating progressive ATV metabolism over time. Median sample storage time was 5.9 years; therefore, it was not unexpected that storage duration was newly associated with analyte concentrations and ratios. Concomitant use of a strong/moderate CYP3A drug inhibitor, or amiodarone itself, was associated with higher ATV concentrations and lower 2‐OH ATV/ATV and 2‐OH ATV L/ATV L ratios, reflecting inhibition of CYP3A‐mediated hydroxylation. Smoking was newly associated with reduced hydroxylation and increased lactonisation ratios. Overall, the proportion of variability explained was modest (R^2^ values of 0.14–0.24). Reassuringly, the secondary analysis models constructed using stepwise covariate selection were highly homologous to the main results (supplementary Table S4).

**Table 3:** Multivariable model results for atorvastatin (ATV) analyte levels

Model characteristic	ATV			2‐OH ATV			ATV L			2‐OH ATV L		
Variable	B (SE)	I	*P*‐value	B (SE)	I	*P*‐value	B (SE)	I	*P*‐value	B (SE)	I	*P*‐value
ATV dose	0.153 (0.058)	0.68	.0084	0.212 (0.048)	1	.000012	0.222 (0.064)	1	.00052	0.215 (0.051)	1	.000023
Sex (F *vs* M)	−0.123 (0.034)	1	.00038	‐	‐	‐	−0.097 (0.038)	0.6	.010	‐	‐	‐
Age	0.004 (0.001)	1	.0016	0.007 (0.001)	1	6.30x10^–8^	‐	‐	‐	0.006 (0.001)	1	3.80x10^–7^
Body mass index	‐	‐	‐	−0.006 (0.002)	0.82	.0047	−0.006 (0.003)	0.36	.016	−0.006 (0.002)	0.8	.0059
Smoking	‐	‐	‐	−0.076 (0.028)	0.64	.0066	‐	‐	‐	‐	‐	‐
Diabetes mellitus	‐	‐	‐	0.079 (0.031)	0.37	.011	‐	‐	‐	‐	‐	‐
Chronic kidney disease	‐	‐	‐	0.111 (0.051)	0.05	.030	‐	‐	‐	‐	‐	‐
Hepatic disease	‐	‐	‐	‐	‐	‐	‐	‐	‐	−0.289 (0.134)	0.16	.032
Loop diuretic	0.099 (0.040)	0.53	.012	0.074 (0.033)	0.06	.025	0.121 (0.043)	0.81	.0044	0.084 (0.035)	0.28	.016
Proton pump inhibitor	0.093 (0.030)	1	.0016	0.062 (0.024)	0.55	.010	0.139 (0.033)	1	.000022	0.106 (0.026)	1	.000041
CYP3A inhibitor	0.166 (0.076)	0.18	.029	‐	‐	‐	‐		‐	‐	‐	‐
Amiodarone	0.282 (0.129)	0.1	.029	‐	‐	‐	0.320 (0.142)	0.18	.024	‐	‐	‐
Sample storage duration	‐	‐	‐	−0.0001 (0.00003)	1	1.14x10^–6^	0.0002 (0.00004)	1	4.00x10^–9^	0.0001 (0.00003)	1	1.44x10^–6^
Time since last ATV	−0.001 (0.0001)	1	<2.0x10^–16^	−0.0007 (0.0001)	1	<2.0x10^–16^	−0.0007 (0.0001)	1	3.15x10^–7^	−0.0006 (0.0001)	1	2.00x10^–8^
Num of models averaged	7			13			8			4		
R^2^	0.17			0.24			0.18			0.21		

**Table 4:** Multivariable model results for atorvastatin (ATV) analyte ratios or sum total

Model characteristic	2‐OH ATV/ATV			2‐OH ATV L/ATV L			ATV L/ATV			TOTAL		
Variable	B (SE)	I	*P*‐value	B (SE)	I	*P*‐value	B (SE)	I	*P*‐value	B (SE)	I	*P*‐value
ATV dose	‐	‐	‐	‐	‐	‐	‐	‐	‐	0.215 (0.046)	1	3.70x10^–6^
Sex (F *vs* M)	0.124 (0.022)	1	<2.0x10^–16^	0.090 (0.022)	1	.000038	‐	‐	‐	‐	‐	‐
Age	0.002 (0.0009)	0.41	.013	0.003 (0.001)	1	.00050	‐	‐	‐	0.005 (0.001)	1	3.91x10^–6^
Body mass index	−0.004 (0.002)	0.39	.013	‐	‐	‐	‐	‐	‐	−0.005 (0.002)	0.65	.0061
Smoking	−0.067 (0.022)	0.89	.0020	−0.058 (0.021)	0.86	.0049	0.102 (0.021)	1	6.90x10^–7^	‐	‐	‐
Diabetes mellitus	‐	‐	‐	‐	‐	‐	−0.055 (0.024)	0.35	.020	‐	‐	‐
Aspirin	‐	‐	‐	0.096 (0.040)	0.33	.016	‐	‐	‐	‐	‐	‐
P2Y_12_ inhibitor	0.076 (0.026)	0.87	.0035	0.077 (0.026)	0.76	.0031	−0.055 (0.026)	0.11	.036	‐		‐
ACEI/ARB	0.068 (0.024)	0.07	.0052	0.058 (0.024)	0.06	.015	‐	‐	‐	‐	‐	‐
Loop diuretic	‐	‐	‐	‐	‐	‐	‐	‐	‐	0.093 (0.032)	1	.0039
Proton pump inhibitor	‐	‐	‐	‐	‐	‐	0.046 (0.019)	0.41	.014	0.087 (0.024)	1	.00023
CYP3A inhibitor	−0.144 (0.048)	1	.0025	−0.120 (0.047)	0.46	.011	‐	‐	‐	‐	‐	‐
Amiodarone	−0.186 (0.083)	0.28	.025	−0.208 (0.082)	0.43	.011	‐	‐	‐	‐	‐	‐
Sample storage duration	−0.0001 (0.00002)	1	1.00x10^–7^	−0.00007 (0.00002)	1	.00087	0.0002 (0.00002)	1	<2.0x10^–16^	‐	‐	‐
Time since last ATV	0.0002 (0.00008)	1	.0060	0.0001 (0.00008)	1	.21	0.0002 (0.00008)	1	.0047	−0.0008 (0.0001)	1	<2.0x10^–16^
Num of models averaged	10			12			5			2		
R^2^	0.18			0.14			0.24			0.21		

I = importance; SE = standard error. Total = ATV + 2‐OH ATV + ATV L + 2‐OH ATV L.

All subsets linear regression was performed using the (maximum) model containing all considered covariates for each endpoint, limited by the requirement that all subsetted models include time since last ATV dose. For each endpoint, regression models were ranked according to BIC, and those with BIC < 2 from the lowest ranked model were averaged to produce the averaged coefficients and standard errors of the final model, displayed in these tables. The relative importance of each covariate included in the final model for a given endpoint was assessed by summing the BIC‐derived model weight of all models that underwent model averaging and contain that covariate (1 indicates high importance). *P*‐values are provided for reference only; they were not used during covariate selection.

ACEI/ARB, angiotensin‐converting enzyme inhibitor/angiotensin II receptor blocker

**Figure 2:**
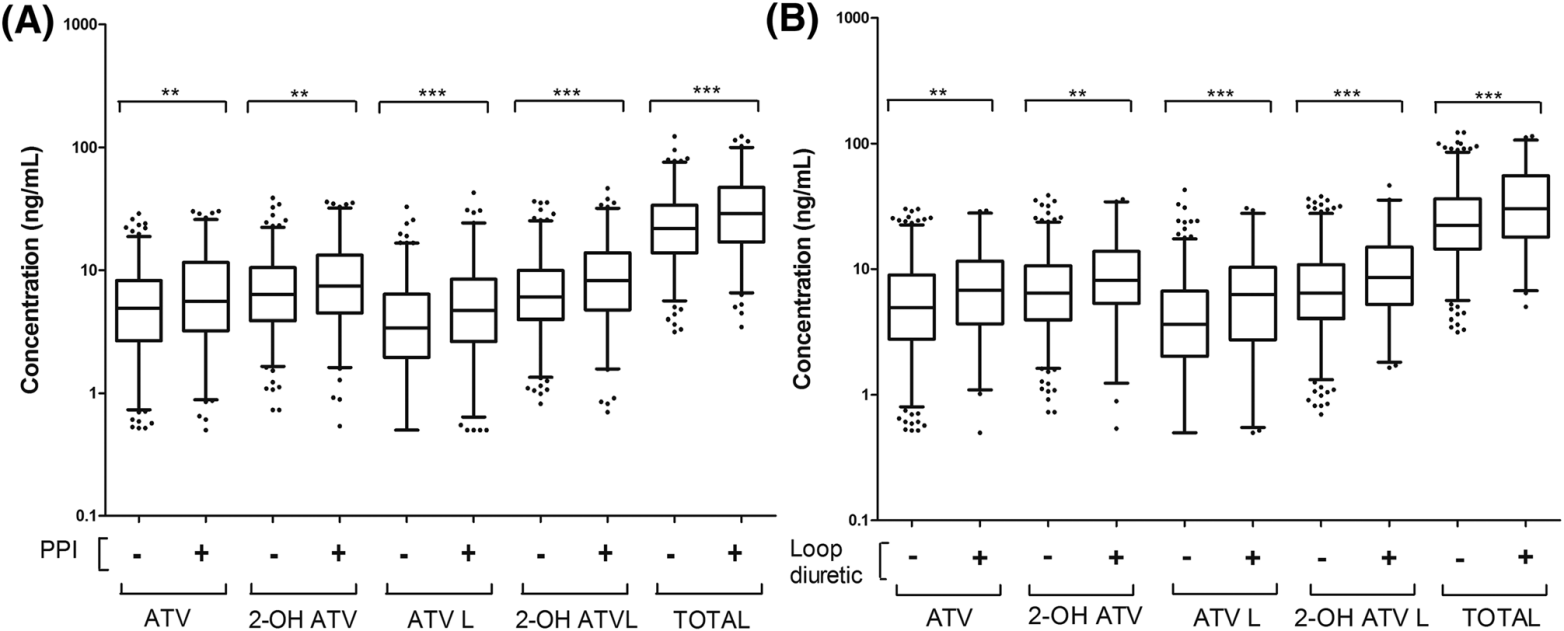
Box and whisker plots of proton pump inhibitor or loop diuretic use on atorvastatin (ATV) analyte levels. The impact of PPI (A) and loop diuretic (B) use. Boxes represent the median (IQR), and whiskers delineate the 2.5% and 97.5% percentiles. Statistical significance was tested here using the Mann–Whitney test on the unadjusted untransformed concentration values (ng/mL); *P*‐value = * <.05; ** <.01; *** <.001. All identified associations are shown, except for the association between PPI use and the ATV L/ATV ratio

The multivariable models suggested novel drug–drug interactions. PPIs and loop diuretics were both associated with higher concentrations of ATV analytes and their total (Figure 2), with relative importance values ranging from 0.55–1.00 and 0.06–1.00, respectively. P2Y_12_ inhibitor use was associated with increased hydroxylation and potentially reduced ATV lactonisation (importance 0.11–0.87). Although, aspirin and ACEI/ARBs were also included in the hydroxylation ratios, their relative importance was low.

### 3.1 Sensitivity analyses

When outliers were removed, all identified associations were significant for smoking and PPIs, and the majority for loop diuretics. The single association between aspirin and increased ATV L hydroxylation remained, and P2Y_12_ inhibitor use (99% on clopidogrel, 1% on prasugrel) was still associated with reduced ATV lactonisation. However, the links between P2Y_12_ inhibitors or ACEI/ARBs and the 2 hydroxylation ratios became insignificant (supplementary Tables S5 and S6).

Within the cohort, of those patients on a loop diuretic at V2, 90% were taking furosemide and 10% bumetanide. For patients taking a PPI at V2, 58% were taking https://www.guidetopharmacology.org/GRAC/LigandDisplayForward?ligandId=7208, 38% https://www.guidetopharmacology.org/GRAC/LigandDisplayForward?ligandId=4279, and 4% another PPI. The vast majority of identified associations remained when furosemide was analysed in place of the loop diuretic drug class, indicating that furosemide principally underlay the associations. Both omeprazole and lansoprazole remained predominantly associated with the relevant endpoints compared to no PPI use, potentially suggestive of a class effect (supplementary Table S7).

### 3.2 Genotype‐stratified analyses

Overall, the evidence favouring potential novel associations between ATV analytes/ratios with PPIs and loop diuretics was considered strongest, and so were explored further in genotype‐stratified analyses (Table 5). Interestingly, the association between PPIs and ATV analytes appeared restricted to *CYP2C19* RM/UM patients, with significant interaction *P*‐values (PPI*CYP2C19 RM/UM *vs* non‐RM/UM) for the lactone analytes and sum analyte total (Table 5). Stratified analysis suggested a possible influence of *ABCG2* rs2231142 on the effect of loop diuretics on ATV analyte levels, but formal interaction testing was insignificant (Table 5).

**Table 5:** The impact of genotype on the relationship between loop diuretics or proton pump inhibitors and the endpoints

	Loop diuretic					Proton pump inhibitor						
	*ABCG2* rs2231142 (C421A, Q141K)				Interaction *P*‐value	*CYP2C19*						Interaction *P*‐value
	WT (*n* = 435)		Variant carrier (*n* = 136)			PM/IM (*n* = 152)		EM (*n* = 233)		RM/UM (*n* = 186)		
	On LD (*n* = 74)		On LD (*n* = 22)			On PPI (*n* = 58)		On PPI (*n* = 88)		On PPI (*n* = 83)		
	B (SE)	*P*‐value	B (SE)	*P*‐value		B (SE)	*P*‐value	B (SE)	*P*‐value	B (SE)	*P*‐value	
ATV	0.121 (0.044)	.0066	0.041 (0.086)	.63	.52	0.046 (0.061)	.46	0.052 (0.045)	.25	0.162 (0.050)	.0015	.078
2‐OH ATV	0.072 (0.039)	.068	−0.051 (0.077)	.51	.22	0.032 (0.050)	.52	0.045 (0.038)	.24	0.191 (0.042)	.0050	.16
ATV L	0.149 (0.048)	.0022	0.077 (0.089)	.38	.54	0.084 (0.070)	.23	0.076 (0.050)	.13	0.232 (0.053)	.000018	.025
2‐OH ATV L	0.102 (0.040)	.011	0.044 (0.075)	.55	.52	0.018 (0.057)	.75	0.065 (0.039)	.094	0.199 (0.042)	5.28x10^–6^	.0071
2‐OH ATV/ATV	‐	‐	‐	‐	‐	‐	‐	‐	‐	‐	‐	‐
2‐OH ATV L/ATV L	‐	‐	‐	‐	‐	‐	‐	‐	‐	‐	‐	‐
ATV L/ATV	‐	‐	‐	‐	‐	0.022 (0.040)	.59	0.021 (0.036)	.49	0.069 (0.031)	.047	.24
TOTAL	0.120 (0.036)	.00088	0.013 (0.069)	.85	.31	0.033 (0.051)	.52	0.060 (0.036)	.094	0.162 (0.039)	.000060	.031

Analysis was conducted limited to those endpoints for which loop diuretic or PPI had been included in the main analysis multivariable model. *CYP2C19* poor and intermediate metabolisers were grouped together because there were only 10 patients with the poor metaboliser (*2/*2) genotype, and *CYP2C19* rapid and ultra‐rapid metabolisers were combined as there were only 24 ultra‐rapid metabolisers (*17/*17). The genotype groups for *CYP2C19* considered in interaction testing were rapid/ultra‐rapid metaboliser (RM/UM) *vs* non‐RM/UM.

## 4 DISCUSSION

The main findings of this study were: confirmation of the impact of several clinical factors (age, sex, time since ATV administration and concomitant use of CYP3A‐inhibiting drugs^4^, ^18^) on exposure to ATV, novel extension of these associations to other ATV metabolites and/or analyte ratios, and identification of new factors associated with ATV analyte exposures including smoking, PPIs, and loop diuretics with the latter attributable principally to furosemide. Moreover, a putative interaction between PPIs and *CYP2C19* genotype on ATV analyte levels was observed. Overall, however, the proportion of observed variability explained was modest.

### 4.1 Clinical factors

Women had lower ATV and ATV L concentrations but increased 2‐OH ATV/ATV and 2‐OH ATV L/ATV L ratios, in keeping with the known ~2‐fold increased CYP3A4 protein levels^27^ and increased CYP3A activity^28^ in female compared to male livers. Increasing age was associated with higher concentrations of ATV analytes, in keeping with previous findings.^29^ Some,^28^, ^30^ but not all^31^ studies have found that CYP3A activity does not change with normal human ageing. Thus, the increased hydroxylation ratios seen here with increasing age are more suggestive of reduced clearance of the hydroxylated metabolites. Higher BMI was associated with lower concentrations of all analytes and total, except for parent ATV, in keeping with increased analyte distribution. Interestingly, a previous sparse pharmacokinetics study limited to parent ATV similarly found no association between ATV and BMI.^18^

CKD was not selected in any of the multivariable models except for 2‐OH ATV, where it was associated with higher 2‐OH ATV levels. However, CKD was only present in 1 of the 13 2‐OH ATV models that underwent model averaging and so its relative importance value of 0.05 was negligible. This is in keeping with the observation that <2% of an ATV dose is excreted through the kidneys.^4^ Chronic alcoholic liver disease markedly increases ATV and ATV acid metabolite levels.^4^ Nevertheless, hepatic disease was not prominent here, probably because only 5 participants reported liver disease.

Smoking was most strongly associated with an increase in the ATV L/ATV ratio. Statin lactonisation of the corresponding acid species occurs predominantly via an unstable acyl glucuronide intermediate, which undergoes spontaneous cyclisation to the resultant lactone.^32^ Cigarette smoke induces several CYPs including CYP1A1, 1A2 and 2E1,^33^, ^34^ but it also induces UGTs.^34^, ^35^ ATV lactonisation is mediated by UGT1A3, and to a lesser extent UGT1A1 and 2B7,^6^ and therefore it is plausible that the increased ATV L/ATV ratio observed is attributable to smoking‐mediated UGT induction. In this study, current *vs* noncurrent (previous/never) smoking was compared. Ever smoking (current/previous, *vs* never) was not associated with any endpoint (data not shown), suggesting that the influence of smoking on ATV metabolism is reversible, in keeping with enzyme induction.

### 4.2 Co‐medications

CYP3A drug inhibitors were associated with a higher ATV concentration and lower 2‐OH ATV/ATV and 2‐OH ATV L/ATV L ratios, which was expected as CYP3A4 is the major enzyme responsible for ATV and ATV L hydroxylation.^5^, ^36^ In this study, the low number of patients concurrently on a CYP3A inducer likely accounts for that lack of signal. Amiodarone is a CYP3A inhibitor of unspecified strength and inhibits P‐glycoprotein^37^; cases of muscle toxicity including rhabdomyolysis have been reported in patients on both amiodarone and a statin.^38^, ^39^, ^40^ Importantly, this present study not only confirmed the impact of amiodarone on ATV hydroxylation, but extended the findings to demonstrate that amiodarone also inhibits ATV L hydroxylation, resulting in elevated ATV L exposure.

Several potential highly novel drug–drug interactions were identified in this study. However, based on the sensitivity analyses and total number of ATV analytes affected, loop diuretics and especially PPIs were considered the most robust novel associations. Both PPIs and loop diuretics were associated here with increased plasma concentrations of all ATV analytes and their sum total, including the more myotoxic lactone metabolites.^12^ Nevertheless, the increases in ATV analyte levels were modest; for example, median ATV concentration increased ~14% (4.9 to 5.6 ng/mL) and ~38% (4.9 to 6.8 ng/mL) with PPIs and loop diuretics, respectively.

Interestingly, ATV severe myotoxicity cases involving a possible interaction with a PPI (predominantly omeprazole) have been reported^41^, ^42^, ^43^, ^44^ and myotoxicity is a possible PPI adverse class effect.^43^ Furthermore, it has been suggested that PPIs may modestly boost statin‐mediated LDL‐C reduction, in keeping with elevated intrahepatic statin levels.^45^ Although lansoprazole and omeprazole are substrates and inhibitors of CYP2C19 and CYP3A4,^22^, ^23^, ^46^, ^47^ CYP3A4‐mediated inhibition would not account for the uniform increase in all ATV analytes observed here; possible explanations include an increase in ATV bioavailability secondary to a PPI‐induced rise in gastric pH, or inhibition of an elimination transporter. Given the exploratory finding that the impact of PPIs on ATV analyte levels appeared restricted to *CYP2C19* RM/UM patients, it is also plausible that a PPI *CYP2C19* product(s) could inhibit a transporter involved in ATV (biliary) efflux, such as BCRP or P‐glycoprotein.^48^

A recent *in vitro* evaluation of furosemide identified it as a substrate for OAT1, OAT3, BCRP, OATP1B1 and OATP1B3.^24^ Moreover, furosemide and bumetanide inhibit BRCP *in vitro*, with furosemide being the more potent inhibitor.^24^, ^25^ Although formal interaction testing between *ABCG2* rs2231142 (Q141K) and loop diuretics was not significantly associated with analyte levels, these are exploratory analyses with a low power to detect an interaction; for instance, the number on a loop diuretic and 141 K was just 22 (Table 5). Thus, follow up functional studies remain of interest. Alternatively, loop diuretics may increase ATV analyte levels through depleting intravascular volume. Neither *CYP2C19* status nor rs2231142 were associated themselves with ATV levels (not shown).

Overall, the magnitude of the identified PPI and loop diuretic associations were modest and of doubtful clinical importance at the population level. However, in specific individuals already likely to have elevated ATV levels due to other risk factors, such as elderly frail patients with comorbidities and polypharmacy, the additional increase in ATV exposure due to these novel drug interactions could plausibly contribute to incident SAM.

### 4.3 Study limitations

To the best of our knowledge, this study constitutes the largest study of ATV systemic exposure to date, and was conducted in a real‐world patient cohort. Furthermore, the main acid and lactone metabolites of ATV were analysed alongside parent ATV. However, there are limitations to this study. First, time since last ATV administration was estimated. Nevertheless, time since last dose is not accurately recorded in many observational pharmacokinetic studies.^49^ Moreover, the mean sampling time (14 h) in this study is on the low curvature aspect of the analyte concentration‐time profiles^8^ and so the effect of sampling time measurement error is expected to be low.^49^ Overall, time was very strongly associated with the majority of endpoints, and several other covariates (e.g. ATV dose, sex, CYP3A inhibitors) behaved similarly as expected in this study, providing confidence in the results. Second, other potentially relevant clinical covariates, such as grapefruit juice and alcohol consumption, were not recorded, and so could not be tested. Third, variation in certain genes (e.g. *SLCO1B1*) is recognised to directly influence ATV levels. However, clinical factors were prioritised here because of their ready availability in clinical practice.

### 4.4 Conclusions

This study has demonstrated multiple factors including demographics, comorbidities and comedications associated with circulating levels of ATV and its major metabolites. Smoking was newly shown to affect ATV metabolism, and commonly prescribed PPIs and loop diuretics were shown for the first time to be associated with increased ATV analyte levels. As the proportion of observed variability explained was modest, future studies should build on the clinical factors identified here and systematically assess the influence of genomics and other ‐omics factors on ATV exposure. Further investigations are also needed to characterise the clinical relevance of these novel clinical associations by, for example, comparing the specific prevalence of PPIs/loop diuretics in ATV myotoxicity cases to statin‐tolerant controls. Finally, prospective studies that quantify ATV levels in susceptible well‐characterised patients starting ATV, with follow up for incident SAM, would help determine the utility of ATV levels and/or multivariable models for pre‐empting SAM.

## COMPETING INTERESTS

There are no competing interests to declare.

## CONTRIBUTORS

*Designed research:* All authors.

*Performed research:* R.M.T., V.F.

*Analysed data*: R.M.T., A.P.M.

*Drafting of manuscript:* R.M.T.

*Critical revision of the manuscript:* All authors.

All authors have approved the final manuscript.

## Supporting information


**FIGURE S1** The study cohort selection process
**TABLE S1** CYP3A inducers and inhibitors
**TABLE S2** The constituents of the individual models selected for averaging for atorvastatin analyte levels in the main analysis
**TABLE S3** The constituents of the individual models selected for averaging for atorvastatin analyte ratios and the analyte sum total in the main analysis
**TABLE S4** The characteristics of the models constructed by multivariable linear regression using stepwise covariate selection (secondary analysis)
**TABLE S5** The association between identified comedications or smoking and atorvastatin analyte levels in multivariable linear regression excluding outliers (sensitivity analysis)
**TABLE S6** The association between identified comedications or smoking and atorvastatin analyte ratios and their sum total in multivariable linear regression excluding outliers (sensitivity analysis)
**TABLE S7** The association between specific comedications and atorvastatin analyte endpoints in multivariable linear regression (sensitivity analysis)

## Data Availability

The data that support the findings of this study are available from the corresponding author upon reasonable request.
