# Supplementary material for: Investigating the clinical factors and comedications associated with circulating levels of atorvastatin and its major metabolites in secondary prevention
Source: Br J Clin Pharmacol. 2020 Jan 4;86(1):62–74. doi: 10.1111/bcp.14133 (PMC6983514; doi:10.1111/bcp.14133)
Supplement: Supplementary file 1 — FIGURE S1 The study cohort selection process TABLE S1 CYP3A inducers and inhibitors TABLE S2 The constituents of the individual models selected for averaging for atorvastatin analyte levels in the main analysis TABLE S3 The constituents of the individual models selected for averaging for atorvastatin analyte ratios and the analyte sum total in the main analysis TABLE S4 The characteristics of the models constructed by multivariable linear regression using stepwise covariate selection (secondary analysis) TABLE S5 The association between identified comedications or smoking and atorvastatin analyte levels in multivariable linear regression excluding outliers (sensitivity analysis) TABLE S6 The association between identified comedications or smoking and atorvastatin analyte ratios and their sum total in multivariable linear regression excluding outliers (sensitivity analysis) TABLE S7 The association between specific comedications and atorvastatin analyte endpoints in multivariable linear regression (sensitivity analysis) [file BCP-86-62-s001.docx]

# Supporting Information to: Investigating the clinical factors and co-medications associated with circulating levels of atorvastatin and its major metabolites in secondary prevention

# Supporting Methods

## Clinical variables

20 clinical variables were used in the multivariable regression analyses, including the presence of any co-medication at V2 listed by the US Food and Drug Administration (FDA) (1) as being a CYP3A-inducer, or a strong/moderate CYP3A-inhibitor (Table S1).

Table S1 CYP3A inducers and inhibitors

| **CYP3A inducers** | **CYP3A strong/moderate inhibitors** |
| --- | --- |
| **Detected in at least one PhACS participant** | |
| carbamazepine | ciclosporin |
| phenytoin | cimetidine |
| - | ciprofloxacin |
| - | clarithromycin |
| - | diltiazem |
| - | dronedarone |
| - | erythromycin |
| - | fluconazole |
| - | itraconazole |
| - | verapamil |
| **Available in UK but not detected in PhACS cohort^1^** | |
| bosentan | aprepitant |
| efavirenz | boceprevir |
| enzalutamide | clotrimazole |
| etravirine | cobicistat |
| mitotane | idelalisib |
| modafinil | imatinib |
| phenobarbitol | ketoconazole |
| rifampicin | luvoxamine |
| rufinamide | posaconazole |
| St John’s wort | voriconazole |
| - | Ritonavir alone or in combination with other antivirals (elvitegravir, indinavir, lopinavir, paritaprevir, saquinavir, teleprevir, tipranavir) |

This list of CYP3A-modifying drugs is taken from the US Food and Drug Administration (1). Strong and moderate CYP3A inhibitors are drugs that increase the area under the concentration-time curve (AUC) of sensitive index substrates ≥5-fold, and ≥2 to <5-fold, respectively (1). ^1^ = grapefruit juice and crizotinib are two FDA listed strong/moderate CYP3A inhibitors. However, it was not possible to detect their use in the PhACS study because the study closed in 2013 before crizotinib was licensed in the UK, and food consumption was not specifically captured in the case report form.

# Supporting Results

This section provides supplementary results pertaining to:

- The study cohort selection flow chart (Figure S1);
- The covariates and model rank (BIC, AICc) of the individual models that were selected for model averaging to produce the main analysis multivariable linear regression model for each endpoint (Tables S2 and S3);
- The covariates selected using multivariable linear regression with stepwise covariate selection for comparison with the main analysis models (secondary analysis Table S4);
- The impact of excluding participants with extreme endpoint values (outside the mean ± 2 SDs) on the identified associations between co-medications or smoking and the endpoints (sensitivity analysis Tables S5 and S6);
- The associations between specific drugs (furosemide, omeprazole, lansoprazole), rather than their drug class as a whole, with the endpoints (sensitivity analysis Table S7).

Figure S1 The study cohort selection process


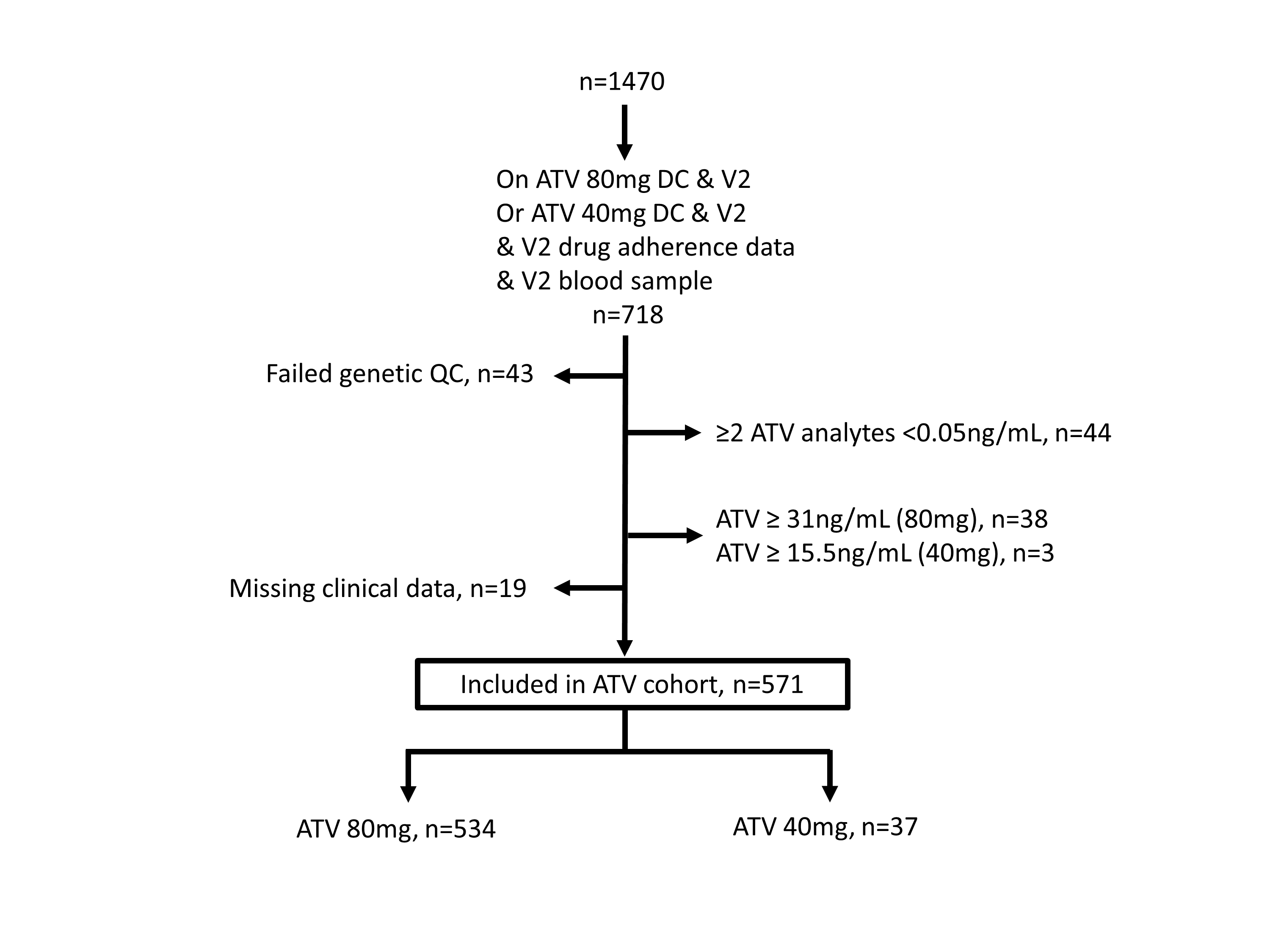


DC = discharge from index hospitalisation; QC = quality control; V2 = visit 2 (month one follow up).

Table S2 The constituents of the individual models selected for averaging for ATV analyte levels in the main analysis

| **Model** | **Variable** | | | | | | | | | | | | | | **Model rank** | |
| --- | --- | --- | --- | --- | --- | --- | --- | --- | --- | --- | --- | --- | --- | --- | --- | --- |
| **ATV** | **ATV Dose** | **Sex** | **Age** | **BMI** | **Smoking** | **Diabetes** | **CKD** | **Hepatic disease** | **Loop diuretic** | **PPI** | **CYP3A inhibitor** | **Amiodarone** | **Sample storage duration** | **Time since last ATV** | **AICc** | **BIC** |
| 1 | **✓** | **✓** | **✓** |  |  |  |  |  | **✓** | **✓** |  |  |  | **✓** | 383.09 | 417.62 |
| 2 |  | **✓** | **✓** |  |  |  |  |  |  | **✓** |  |  |  | **✓** | 391.85 | 417.78 |
| 3 | **✓** | **✓** | **✓** |  |  |  |  |  |  | **✓** |  |  |  | **✓** | 387.61 | 417.84 |
| 4 |  | **✓** | **✓** |  |  |  |  |  | **✓** | **✓** |  |  |  | **✓** | 388.33 | 418.56 |
| 5 | **✓** | **✓** | **✓** |  |  |  |  |  | **✓** | **✓** |  | **✓** |  | **✓** | 380.30 | 419.11 |
| 6 | **✓** | **✓** | **✓** |  |  |  |  |  | **✓** | **✓** | **✓** |  |  | **✓** | 380.36 | 419.17 |
| 7 | **✓** | **✓** | **✓** |  |  |  |  |  |  | **✓** | **✓** |  |  | **✓** | 384.85 | 419.37 |
| **2-OH ATV** | **ATV Dose** | **Sex** | **Age** | **BMI** | **Smoking** | **Diabetes** | **CKD** | **Hepatic disease** | **Loop diuretic** | **PPI** | **CYP3A4 inhibitor** | **Amiodarone** | **Sample storage duration** | **Time since last ATV** | **AICc** | **BIC** |
| 1 | **✓** |  | **✓** | **✓** | **✓** | **✓** |  |  |  | **✓** |  |  | **✓** | **✓** | 170.98 | 214.06 |
| 2 | **✓** |  | **✓** | **✓** | **✓** |  |  |  |  | **✓** |  |  | **✓** | **✓** | 175.62 | 214.42 |
| 3 | **✓** |  | **✓** | **✓** | **✓** | **✓** |  |  |  |  |  |  | **✓** | **✓** | 175.63 | 214.43 |
| 4 | **✓** |  | **✓** | **✓** | **✓** |  |  |  |  |  |  |  | **✓** | **✓** | 179.92 | 214.44 |
| 5 | **✓** |  | **✓** |  |  |  |  |  |  | **✓** |  |  | **✓** | **✓** | 185.11 | 215.34 |
| 6 | **✓** |  | **✓** | **✓** |  | **✓** |  |  |  | **✓** |  |  | **✓** | **✓** | 176.66 | 215.46 |
| 7 | **✓** |  | **✓** | **✓** |  |  |  |  |  | **✓** |  |  | **✓** | **✓** | 181.12 | 215.64 |
| 8 | **✓** |  | **✓** | **✓** | **✓** |  |  |  | **✓** |  |  |  | **✓** | **✓** | 176.86 | 215.67 |
| 9 | **✓** |  | **✓** | **✓** |  |  |  |  |  |  |  |  | **✓** | **✓** | 185.59 | 215.82 |
| 10 | **✓** |  | **✓** |  |  |  |  |  |  |  |  |  | **✓** | **✓** | 189.96 | 215.89 |
| 11 | **✓** |  | **✓** |  | **✓** |  |  |  |  | **✓** |  |  | **✓** | **✓** | 181.39 | 215.91 |
| 12 | **✓** |  | **✓** | **✓** |  | **✓** |  |  |  |  |  |  | **✓** | **✓** | 181.48 | 216.00 |
| 13 | **✓** |  | **✓** | **✓** | **✓** |  | **✓** |  |  | **✓** |  |  | **✓** | **✓** | 172.93 | 216.01 |
| **ATV L** | **ATV Dose** | **Sex** | **Age** | **BMI** | **Smoking** | **Diabetes** | **CKD** | **Hepatic disease** | **Loop diuretic** | **PPI** | **CYP3A4 inhibitor** | **Amiodarone** | **Sample storage duration** | **Time since last ATV** | **AICc** | **BIC** |
| 1 | **✓** |  |  |  |  |  |  |  | **✓** | **✓** |  |  | **✓** | **✓** | 499.47 | 529.71 |
| 2 | **✓** | **✓** |  |  |  |  |  |  | **✓** | **✓** |  |  | **✓** | **✓** | 495.19 | 529.71 |
| 3 | **✓** | **✓** |  | **✓** |  |  |  |  | **✓** | **✓** |  |  | **✓** | **✓** | 491.31 | 530.11 |
| 4 | **✓** |  |  | **✓** |  |  |  |  | **✓** | **✓** |  |  | **✓** | **✓** | 496.11 | 530.63 |
| 5 | **✓** |  |  |  |  |  |  |  |  | **✓** |  |  | **✓** | **✓** | 504.91 | 530.84 |
| 6 | **✓** | **✓** |  | **✓** |  |  |  |  | **✓** | **✓** |  | **✓** | **✓** | **✓** | 488.00 | 531.08 |
| 7 | **✓** | **✓** |  |  |  |  |  |  | **✓** | **✓** |  | **✓** | **✓** | **✓** | 492.32 | 531.12 |
| 8 | **✓** | **✓** |  |  |  |  |  |  |  | **✓** |  |  | **✓** | **✓** | 501.09 | 531.33 |
| **2-OH ATV L** | **ATV Dose** | **Sex** | **Age** | **BMI** | **Smoking** | **Diabetes** | **CKD** | **Hepatic disease** | **Loop diuretic** | **PPI** | **CYP3A4 inhibitor** | **Amiodarone** | **Sample storage duration** | **Time since last ATV** | **AICc** | **BIC** |
| 1 | **✓** |  | **✓** | **✓** |  |  |  |  |  | **✓** |  |  | **✓** | **✓** | 238.53 | 273.06 |
| 2 | **✓** |  | **✓** | **✓** |  |  |  |  | **✓** | **✓** |  |  | **✓** | **✓** | 234.76 | 273.57 |
| 3 | **✓** |  | **✓** |  |  |  |  |  |  | **✓** |  |  | **✓** | **✓** | 244.02 | 274.25 |
| 4 | **✓** |  | **✓** | **✓** |  |  |  | **✓** |  | **✓** |  |  | **✓** | **✓** | 235.92 | 274.72 |

For each endpoint, all subsets linear regression was performed on the (maximum) model containing all considered covariates, limited by the requirement that all subsetted models include the time since last ATV dose covariate. For each endpoint, only those models that had a BIC<2 from the lowest ranked model are displayed, because model averaging was restricted to the covariates within this cohort of models to minimise the incorporation of weakly associated covariates into the final model. The AICc is also provided for each subsetted model for reference.

Table S3 The constituents of the individual models selected for averaging for ATV analyte ratios and the analyte sum total in the main analysis

| **Model** | **Variable** | | | | | | | | | | | | | | | **Model rank** | |
| --- | --- | --- | --- | --- | --- | --- | --- | --- | --- | --- | --- | --- | --- | --- | --- | --- | --- |
| **2-OH ATV/ ATV** | **ATV Dose** | **Sex** | **Age** | **BMI** | **Smoking** | **Diabetes** | **Aspirin** | **P2Y_12_ inhibitor** | **ACEI/ ARB** | **Loop diuretic** | **PPI** | **CYP3A inhibitor** | **Amiodarone** | **Sample storage duration** | **Time since last ATV** | **AICc** | **BIC** |
| 1 |  | **✓** |  | **✓** | **✓** |  |  | **✓** |  |  |  | **✓** |  | **✓** | **✓** | -159.52 | -120.72 |
| 2 |  | **✓** | **✓** |  | **✓** |  |  | **✓** |  |  |  | **✓** |  | **✓** | **✓** | -159.29 | -120.49 |
| 3 |  | **✓** |  |  | **✓** |  |  | **✓** |  |  |  | **✓** |  | **✓** | **✓** | -154.89 | -120.37 |
| 4 |  | **✓** | **✓** |  |  |  |  | **✓** |  |  |  | **✓** |  | **✓** | **✓** | -154.33 | -119.81 |
| 5 |  | **✓** | **✓** | **✓** | **✓** |  |  | **✓** |  |  |  | **✓** |  | **✓** | **✓** | -162.36 | -119.28 |
| 6 |  | **✓** | **✓** |  | **✓** |  |  | **✓** |  |  |  | **✓** | **✓** | **✓** | **✓** | -162.16 | -119.08 |
| 7 |  | **✓** |  | **✓** | **✓** |  |  | **✓** |  |  |  | **✓** | **✓** | **✓** | **✓** | -162.08 | -119.00 |
| 8 |  | **✓** |  | **✓** | **✓** |  |  |  | **✓** |  |  | **✓** |  | **✓** | **✓** | -157.76 | -118.96 |
| 9 |  | **✓** |  |  | **✓** |  |  | **✓** |  |  |  | **✓** | **✓** | **✓** | **✓** | -157.73 | -118.93 |
| 10 |  | **✓** |  |  | **✓** |  |  |  |  |  |  | **✓** | **✓** | **✓** | **✓** | -153.33 | -118.81 |
| **2-OH ATV L /ATV L** | **ATV Dose** | **Sex** | **Age** | **BMI** | **Smoking** | **Diabetes** | **Aspirin** | **P2Y_12_ inhibitor** | **ACEI/ ARB** | **Loop diuretic** | **PPI** | **CYP3A4 inhibitor** | **Amiodarone** | **Sample storage duration** | **Time since last ATV** | **AICc** | **BIC** |
| 1 |  | **✓** | **✓** |  | **✓** |  |  | **✓** |  |  |  | **✓** |  | **✓** | **✓** | -162.47 | -123.66 |
| 2 |  | **✓** | **✓** |  | **✓** |  |  | **✓** |  |  |  |  |  | **✓** | **✓** | -157.90 | -123.38 |
| 3 |  | **✓** | **✓** |  | **✓** |  |  | **✓** |  |  |  |  | **✓** | **✓** | **✓** | -161.72 | -122.91 |
| 4 |  | **✓** | **✓** |  | **✓** |  | **✓** | **✓** |  |  |  | **✓** |  | **✓** | **✓** | -165.69 | -122.61 |
| 5 |  | **✓** | **✓** |  | **✓** |  |  | **✓** |  |  |  | **✓** | **✓** | **✓** | **✓** | -165.61 | -122.53 |
| 6 |  | **✓** | **✓** |  |  |  |  | **✓** |  |  |  | **✓** |  | **✓** | **✓** | -156.80 | -122.28 |
| 7 |  | **✓** | **✓** |  |  |  |  | **✓** |  |  |  |  |  | **✓** | **✓** | -152.31 | -122.07 |
| 8 |  | **✓** | **✓** |  | **✓** |  | **✓** |  |  |  |  |  | **✓** | **✓** | **✓** | -160.87 | -122.07 |
| 9 |  | **✓** | **✓** |  | **✓** |  | **✓** |  |  |  |  | **✓** | **✓** | **✓** | **✓** | -164.98 | -121.90 |
| 10 |  | **✓** | **✓** |  | **✓** |  | **✓** | **✓** |  |  |  |  |  | **✓** | **✓** | -160.59 | -121.79 |
| 11 |  | **✓** | **✓** |  | **✓** |  | **✓** |  | **✓** |  |  |  | **✓** | **✓** | **✓** | -164.76 | -121.68 |
| 12 |  | **✓** | **✓** |  | **✓** |  |  |  |  |  |  |  | **✓** | **✓** | **✓** | -156.20 | -121.68 |
| **ATV L/ATV** | **ATV Dose** | **Sex** | **Age** | **BMI** | **Smoking** | **Diabetes** | **Aspirin** | **P2Y_12_ inhibitor** | **ACEI/ ARB** | **Loop diuretic** | **PPI** | **CYP3A4 inhibitor** | **Amiodarone** | **Sample storage duration** | **Time since last ATV** | **AICc** | **BIC** |
| 1 |  |  |  |  | **✓** |  |  |  |  |  |  |  |  | **✓** | **✓** | -111.15 | -89.52 |
| 2 |  |  |  |  | **✓** |  |  |  |  |  | **✓** |  |  | **✓** | **✓** | -115.17 | -89.24 |
| 3 |  |  |  |  | **✓** | **✓** |  |  |  |  |  |  |  | **✓** | **✓** | -114.59 | -88.66 |
| 4 |  |  |  |  | **✓** | **✓** |  |  |  |  | **✓** |  |  | **✓** | **✓** | -118.53 | -88.30 |
| 5 |  |  |  |  | **✓** |  |  | **✓** |  |  |  |  |  | **✓** | **✓** | -113.53 | -87.60 |
| **TOTAL** | **ATV Dose** | **Sex** | **Age** | **BMI** | **Smoking** | **Diabetes** | **Aspirin** | **P2Y_12_ inhibitor** | **ACEI/ ARB** | **Loop diuretic** | **PPI** | **CYP3A4 inhibitor** | **Amiodarone** | **Sample storage duration** | **Time since last ATV** | **AICc** | **BIC** |
| 1 | **✓** |  | **✓** | **✓** |  |  |  |  |  | **✓** | **✓** |  |  |  | **✓** | 136.87 | 171.40 |
| 2 | **✓** |  | **✓** |  |  |  |  |  |  | **✓** | **✓** |  |  |  | **✓** | 142.40 | 172.63 |

Total = ATV + 2-OH ATV + ATV L + 2-OH ATV L

For each endpoint, all subsets linear regression was performed on the (maximum) model containing all considered covariates, limited by the requirement that all subsetted models include the time since last ATV dose covariate. For each endpoint, only those models that had a BIC<2 from the lowest ranked model are displayed, because model averaging was restricted to the covariates within this cohort of models to minimise the incorporation of weakly associated covariates into the final model. The AICc is also provided for each subsetted model for reference.

Table S4 The characteristics of the models constructed by multivariable linear regression using stepwise covariate selection (secondary analysis)

| **Model characteristic** | **ATV** | **2-OH ATV** | **ATV L** | **2-OH ATV L** | **2-OH ATV/ATV** | **2-OH ATV L/ATV L** | **ATV L/ATV** | **TOTAL** |
| --- | --- | --- | --- | --- | --- | --- | --- | --- |
| ATV Dose | **✓** | **✓** | **✓** | **✓** | - | - | - | **✓** |
| Sex (M vs F) | **✓** | - | **✓** | - | **✓** | **✓** | - | - |
| Age | **✓** | **✓** | - | **✓** | **✓** | **✓** | - | **✓** |
| BMI | - | **✓** | **✓** | **✓** | **✓** | - | - | **✓** |
| Smoking | - | **✓** | - | - | **✓** | **✓** | **✓** | - |
| Diabetes mellitus | - | **✓** | - | - | - | - | **✓** | - |
| CKD | - | **X** | - | - | - | - | - | - |
| Hepatic disease | - | - | - | **✓** | - | - | - | - |
| Aspirin | - | - | - | - | - | **✓** | - | - |
| P2Y_12_ inhibitor | - | - | - | - | **✓** | **✓** | **X** | - |
| ACEI/ARB | - | - | - | - | **X** | **X** | - | - |
| Loop diuretic | **✓** | **X** | **✓** | **✓** | - | - | - | **✓** |
| Proton pump inhibitor | **✓** | **✓** | **✓** | **✓** | - | - | **✓** | **✓** |
| CYP3A inhibitor | **✓** | - | - | - | **✓** | **✓** | - | - |
| Amiodarone | **✓** | - | **✓** | - | **✓** | **✓** | - | - |
| Sample storage duration | - | **✓** | **✓** | **✓** | **✓** | **✓** | **✓** | - |
| Time since last ATV | **✓** | **✓** | **✓** | **✓** | **✓** | **✓** | **✓** | **✓** |

**✓ =** selected in both the main analysis and secondary analysis models; **X** = present in the main analysis model but not the secondary analysis model. No covariate was selected in a secondary analysis model but was not in the associated main analysis model.

The main analysis models were produced by all subsets linear regression, with the models ranked by BIC and those models with BIC Δ<2 from the lowest ranked model underwent model averaging. The secondary analysis models were produced by multivariable linear regression using stepwise covariate selection based on the probability of F (the unselected covariate with the lowest F probability, provided it was <0.05, was selected, whilst previously selected covariates whose F probability becomes >0.10 are then excluded).

Table S5 The association between identified co-medications or smoking and ATV analyte levels in multivariable linear regression excluding outliers (sensitivity analysis)

| **Covariate** | **ATV (n=547)** | | **2-OH ATV (n=555)** | | **ATV L (n=537)** | | **2-OH ATV L (n=545)** | |
| --- | --- | --- | --- | --- | --- | --- | --- | --- |
|  | **B (SE)** | **p-value** | **B (SE)** | **p-value** | **B (SE)** | **p-value** | **B (SE)** | **p-value** |
| Smoking | - | - | -0.069 (0.026) | 0.0093 | - | - | - | - |
| Loop diuretic | 0.073 (0.035) | 0.041 | 0.024 (0.039) | 0.48 | 0.121 (0.039) | 0.0020 | 0.059 (0.031) | 0.059 |
| Proton pump inhibitor | 0.066 (0.026) | 0.013 | 0.058 (0.023) | 0.013 | 0.096 (0.029) | 0.0012 | 0.087 (0.023) | 0.00018 |
| CYP3A4 inhibitor | 0.131 (0.072) | 0.072 | - | - | - | - | - | - |
| Amiodarone | 0.252 (0.114) | 0.028 | - | - | 0.257 (0.136) | 0.060 | - | - |

For smoking and each co-medication selected for inclusion in the final (model-averaged) multivariable model of a given endpoint, the robustness of its association with this endpoint was investigated further by first excluding participants that had a value for that endpoint outside the mean ± two standard deviations, and then re-running the multivariable model. The rationale for this sensitivity analysis was because linear regression can be sensitive to outliers. For each endpoint, only the effect size (coefficient and standard error) and p-value of smoking or the relevant co-medication(s) in the endpoint multivariable model are reported. P<0.05 was taken as significant.

Table S6 The association between identified co-medications or smoking and ATV analyte ratios and their sum total in multivariable linear regression excluding outliers (sensitivity analysis)

| **Covariate** | **2-OH ATV/ATV (n=539)** | | **2-OH ATV L/ATV L (n=546)** | | **ATV L/ATV (n=544)** | | **TOTAL (n=549)** | |
| --- | --- | --- | --- | --- | --- | --- | --- | --- |
|  | **B (SE)** | **p-value** | **B (SE)** | **p-value** | **B (SE)** | **p-value** | **B (SE)** | **p-value** |
| Smoking | -0.060 (0.018) | 0.00090 | -0.064 (0.018) | 0.00033 | 0.071 (0.018) | 0.000089 | - | - |
| Aspirin | - | - | 0.073 (0.038) | 0.031 | - | - | - | - |
| P2Y_12_ inhibitor | 0.020 (0.023) | 0.40 | 0.036 (0.024) | 0.13 | -0.050 (0.023) | 0.032 | - | - |
| ACEI/ARB | 0.025 (0.022) | 0.27 | 0.025 (0.022) | 0.27 | - | - | - | - |
| Loop diuretic | - | - | - | - | - | - | 0.085 (0.030) | 0.0044 |
| Proton pump inhibitor | - | - | - | - | 0.027 (0.017) | 0.11 | 0.074 (0.022) | 0.00069 |
| CYP3A4 inhibitor | -0.139 (0.041) | 0.00075 | -0.134 (0.041) | 0.0012 | - | - | - | - |
| Amiodarone | -0.114 (0.074) | 0.12 | -0.100 (0.074) | 0.18 | - | - | - | - |

For smoking and each co-medication selected for inclusion in the final (model-averaged) multivariable model of a given endpoint, the robustness of its association with this endpoint was investigated further by first excluding participants that had a value for that endpoint outside the mean ± two standard deviations, and then re-running the multivariable model. The rationale for this sensitivity analysis was because linear regression can be sensitive to outliers. For each endpoint, only the effect size (coefficient and standard error) and p-value of smoking or the relevant co-medication(s) in the endpoint multivariable model are reported. P<0.05 was taken as significant.

Table S7 The association between specific co-medications and ATV analyte endpoints in multivariable linear regression (sensitivity analysis)

| **Endpoint** | **Furosemide (n=86)** | | **Omeprazole (n=87)** | | **Lansoprazole (n=133)** | |
| --- | --- | --- | --- | --- | --- | --- |
|  | **B (SE)** | **p-value** | **B (SE)** | **p-value** | **B (SE)** | **p-value** |
| ATV | 0.104 (0.041) | 0.011 | 0.069 (0.040) | 0.085 | 0.086 (0.035) | 0.013 |
| 2-OH ATV | 0.042 (0.036) | 0.241 | 0.075 (0.034) | 0.027 | 0.046 (0.028) | 0.108 |
| ATV L | 0.142 (0.044) | 0.001 | 0.119 (0.045) | 0.009 | 0.125 (0.039) | 0.001 |
| 2-OH ATV L | 0.090 (0.036) | 0.014 | 0.111 (0.035) | 0.002 | 0.080 (0.031) | 0.011 |
| 2-OH ATV/ATV | - | - | - | - | - | - |
| 2-OH ATV L/ATV L | - | - | - | - | - | - |
| ATV L/ATV | - | - | 0.023 (0.027) | 0.40 | 0.044 (0.023) | 0.054 |
| TOTAL | 0.100 (0.033) | 0.003 | 0.102 (0.032) | 0.002 | 0.067 (0.028) | 0.017 |

The identified associations between PPI or loop diuretic drug classes with the endpoints were investigated further by substituting the drug class for the prevalent drug(s) in each drug class found in the cohort, and then repeating the multivariable linear regression. For each endpoint, all other covariates in the multivariable linear regression model were unchanged from those included in the main analysis final model. For each drug (furosemide, omeprazole, lansoprazole), only those endpoints where the relevant drug class had been included in the endpoint’s main analysis final model were analysed. The comparison group for furosemide, omeprazole and lansoprazole use were PhACS participants not taking any drug from the relevant drug class at V2 (e.g. furosemide vs no loop diuretic). P<0.05 was taken as significant.

# Supporting References

(1) Food and Drug Administration. *Drug Development and Drug Interactions: Table of Substrates, Inhibitors and Inducers*. <https://www.fda.gov/drugs/developmentapprovalprocess/developmentresources/druginteractionslabeling/ucm093664.htm> (2016). Accessed 7 January 2019.
